# Evaluation of endothelial function and subclinical atherosclerosis in patients with HIV infection

**DOI:** 10.1038/s41598-021-97795-2

**Published:** 2021-09-16

**Authors:** F. Arnaiz de las Revillas, V. Gonzalez-Quintanilla, J. A. Parra, E. Palacios, C. Gonzalez-Rico, C. Armiñanzas, M. Gutiérrez-Cuadra, A. Oterino, C. Fariñas-Alvarez, M. C. Fariñas

**Affiliations:** 1 Infectious Diseases Service, Hospital Universitario Marqués de Valdecilla-IDIVAL, University of Cantabria, Av. Valdecilla s/n, 39008 Santander, Spain; 2 Neurology Service, Hospital Universitario Marqués de Valdecilla, Santander, Spain; 3 Radiology Service, Hospital Universitario Marqués de Valdecilla-IDIVAL, University of Cantabria, Santander, Spain; 4 Quality Unit. Hospital Universitario Marqués de Valdecilla-IDIVAL, Santander, Spain

**Keywords:** Infection, Lymphocytes, Cardiology, Risk factors

## Abstract

The aim of this study was to analyse the association between human immunodeficiency virus (HIV) related clinical and analytical parameters and the presence of subclinical atherosclerosis as well as endothelial dysfunction. This was a prospective cohort study of HIV-positive patients who underwent intima media thickness (IMT) determination and coronary artery calcium scoring to determine subclinical atherosclerosis. To detect endothelial dysfunction, the breath holding index, flow-mediated dilation and the concentration of endothelial progenitor cells (EPCs) were measured. Patients with an IMT ≥ 0.9 mm had an average of 559.3 ± 283.34 CD4/μl, and those with an IMT < 0.9 mm had an average of 715.4 ± 389.92 CD4/μl (p = 0.04). Patients with a low calcium score had a significantly higher average CD4 cell value and lower zenith viral load (VL) than those with a higher score (707.7 ± 377.5 CD4/μl vs 477.23 ± 235.7 CD4/μl (p = 0.01) and 7 × 10^4^ ± 5 × 10^4^ copies/ml vs 23.4 × 104 ± 19 × 104 copies/ml (p = 0.02)). The number of early EPCs in patients with a CD4 nadir < 350/µl was lower than that in those with a CD4 nadir ≥ 350 (p = 0.03). In HIV-positive patients, low CD4 cell levels and high VL were associated with risk of developing subclinical atherosclerosis. HIV patients with CD4 cell nadir < 350/µl may have fewer early EPCs.

## Introduction

Highly active antiretroviral treatment (HAART) has allowed patients with human immunodeficiency virus (HIV) infection to have a life expectancy similar to that of the general population, and non-acquired immunodeficiency syndrome (AIDS)-related events are globally more frequent than classic AIDS events in developed countries^1,2^. Thus, cardiovascular disease, which is the leading cause of death in the general world population^3^, presents in people with HIV infection^4^.

Cardiovascular risk prediction functions used in the general population using classical cardiovascular risk factors may be inaccurate and underestimate the risk in HIV-infected patients^5^. New analytical and radiological markers are being investigated to achieve an earlier diagnosis of atherosclerosis that will allow a more accurate selection of patients who need to perform primary cardiovascular prophylaxis^6^. Among radiological tests to determine subclinical atherosclerosis, carotid Doppler ultrasound^7–10^ for the measurement of mean intima media thickness (IMT) and coronary artery calcium score^11–13^ are the most studied in HIV-infected people.

Other radiodiagnosis tests for the early detection of endothelial dysfunction, such as endothelial-dependent vasodilation or flow-mediated dilation (FMD) of the brachial artery^14,15^ and the breath holding index (BHI)^16,17^, have inconclusive data in the HIV population. Circulating endothelial progenitor cells (EPCs) are characterized by their ability to perform endothelial repair^18^. There are few studies that have evaluated EPCs in the context of HIV infection with divergent results. This may be related to the heterogeneity of the methodology of the studies, taking into account the differences between the characteristics of the populations^19–24^.

Due to the potential cardiovascular risk in patients with HIV infection, an early diagnosis of subclinical atherosclerosis and endothelial dysfunction is important to establish preventive interventions, not only in lifestyle change but also pharmacologically if necessary. The detection of clinical and laboratory parameters related to HIV and associated with the appearance of subclinical atherosclerosis and endothelial dysfunction could provide clues about these early stages of atherosclerosis and lead to the performance of diagnostic tests that are more sensitive, specific, safe for the patient and use an easily reproducible technique.

The aim of this study was to analyse in patients with HIV infection whether there is an association between clinical (time of infection, length of HAART) and analytical parameters (total number and nadir of CD4 cells, zenith viral load-VL) and endothelial dysfunction, measured by FMD, BHI and EPCs, as well as with the presence of subclinical atherosclerosis assessed by the coronary artery calcium score and the determination of IMT.

## Results

The patients included had a mean of 15.5 ± 6.9 years of infection duration and 14.1 ± 6.25 years of treatment. The average Charlson index^25^ was significantly higher in men: 1.78 ± 0.4 vs 1.07 ± 0.3 (p = 0.04). Table 1 details the clinical and analytical characteristics of the included patients and their differences by sex.

**Table 1:** Patient characteristics stratified by sex.

	Male Mean (SD) (n = 51)	Female Mean (SD) (n = 26)	*P value*^*a*^
Age (years)	51.8 ± 11.9	47.04 ± 7.8	0.06
Weight (kg)	81.6 ± 11.6	64.8 ± 12.24	< 0.001
BMI (kg/m^2^)	27 ± 3.59	25 ± 4.72	0.07
Abdominal perimeter (cm)	99.59 ± 9.9	96.2 ± 23.2	0.62
SBP (mmHg)	132.49 ± 15.21	122 ± 17.8	0.01
MBP (mmHg)	105.5 ± 11.3	98.6 ± 13.73	0.03
**HIV analytical related parameters**			
Zenith VL (copies/ml)	167,953 ± 196,611	348,236 ± 1,167,861	0.22
Lymphocytes T CD4 (cells/ μl)	906.7 ± 441.9	831.4 ± 538.5	0.53
CD4%	30.3 ± 10.5	33.4 ± 9.7	0.24
CD4/CD8	0.83 ± 0.44	0.94 ± 0.48	0.36
**HIV clinical related parameters**			
Months since diagnosis HIV	197.7 + 80.8	237.2 + 84.7	0.06
HAART (months)	166 + 77.6	178 + 70.8	0.51
**Lipid profile**			
T Col (mg/dl)	174.31 ± 47.16	193.38 ± 54.47	0.13
HDLCol (mg/dl)	46.61 ± 31.40	59.81 ± 17.29	0.002
LDLCol (mg/dl)	105.39 ± 31.40	114.42 ± 37.02	0.29
Triglycerides (mg/dl)	149.92 ± 106.28	140.5 ± 135.09	0.73

*SD* standard deviation, *VL* viral load, *HIV* human immunodeficiency virus, *HAART* highly active antiretroviral therapy, *T Col* total cholesterol, *HDLCol* cholesterol bound to high density lipoproteins, *LDLCol* cholesterol bound to low density lipoproteins, *SBP* systolic blood pressure, *MBP* mean blood pressure.

^a^Two-tailed one-way ANOVA test.

**Figure 1:**
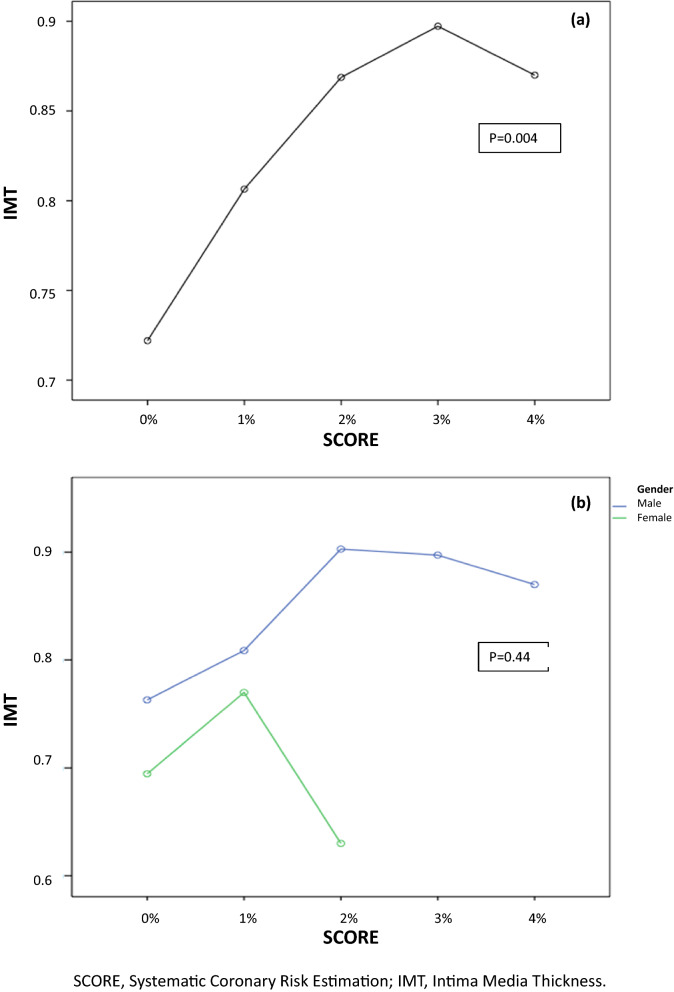
Asociation between IMT and SCORE. (**a**) Asociation between IMT and SCORE in the one-way ANOVA. (**b**) Asociation between IMT and SCORE and sex as independent factors in the two-way ANOVA.

The mean IMT in men was 0.79 ± 0.13 mm, and in women, it was 0.68 ± 0.15 mm (p = 0.002). A significant association was found between IMT and the likelihood of suffering a fatal cardiovascular event in the next 10 years by the systematic coronary risk estimation index (SCORE) using an ANOVA test (p = 0.02) (Fig. 1).

Thirty percent (23/77) of the patients presented a pathological IMT, and in 19.5% (15/77), atheroma plaques were observed at the carotid level. Virological and immunological differences, as well as disparities in other inflammatory biomarkers (ultrasensitive PCR and D dimer), between patients with IMT within normal limits and patients with pathological values are reflected in Table 2.

**Table 2:** Clinical and analytical factors associated with subclinical atherosclerosis in patients with HIV infection.

Characteristics of patients	Intima media thickness			Coronariy calcium score		
n (%) or mean ± SD	< 0,9 mm (*n* = 54)	≥ 0,9 mm (n = 23)	*P* value^a^	0–100 UA (n = 64)	> 100 UA (n = 13)	*P* value^a^
Male, n (%)	31 (57.4)	20 (87.0)	0.01	40/64 (63.0)	11/13 (84.6)11/13)	0.12
Age (years)	47.8 ± 10.4	55.8 ± 9.98	0.02	48.09 ± 9.93	60.7 ± 9.87	0.01
BMI (Kg/m^2^)	25.89 ± 3.76	27.37 ± 4.68	0.14	26.02 ± 3.87	27.85 ± 4.88	0.14
MBP (mmHg)	101.56 ± 12.07	106.95 ± 13.03	0.09	103.10 ± 12.8	103.53 ± 11.5	0.91
**HIV analytical related parameters**						
CD4 (cells/μl)	715.4 ± 389.92	559.3 ± 283.34	0.04	707.7 ± 377.5	477.23 ± 235.7	0.01
CD4%	33.2 ± 9.47	26.9 ± 10.99	0.02	32.0 ± 9.5	28.3 ± 10.6	0.25
CD4/CD8	0.85 ± 0.41	0.74 ± 0.3	0.06	0.88 ± 0.47	0.79 ± 0.4	0.54
Nadir CD4 (cells/μl)	269.5 ± 172.5	236.9 ± 168.2	0.45	345.4 ± 182.9	207.7 ± 148.1	0.04
Zenith VL (copies/ml)	15 × 10^4^ ± 17 × 10^4^	25.9 × 10^4^ ± 8 × 10^4^	0.36	7 × 10^4^ ± 5 × 10^4^	23.4 × 10^4^ ± 19 × 10^4^	0.02
**HIV clinical related parameters**						
Months since diagnosis HIV	206.04 ± 89.52	222.60 ± 68.64	0,43	211.20 ± 84.84	210.36 ± 80.88	0.84
HAART (months)	166.78 ± 80.37	178.47 ± 61.80	0,49	169.53 ± 76.97	173.92 ± 67.84	0.97
**Lipid profile**						
T Col (mg/dl)	185.69 ± 59.7	178.64 ± 46.1	0.57	183.11 ± 52.7	169.15 ± 34.87	0.36
HDLCol (mg/dl)	55.04 ± 21.76	49.37 ± 16.18	0.21	51.03 ± 18.2	51.23 ± 18.08	0.90
LDLCol (mg/dl)	107.68 ± 33.28	110.22 ± 34.5	0.76	110.92 ± 33.91	96.30 ± 29.14	0.15
Tryglicerides (mg/dl)	153.51 ± 54.9	130.82 ± 54.9	0.44	154.18 ± 124.12	110.08 ± 50.73	0.21

*SD* standard deviation, *u-CRP* ultrasensitive C-reactive protein, *VL* viral load, *TCol* total cholesterol, *HDLCol* cholesterol bound to high density lipoproteins, *LDLCol* cholesterol bound to low density lipoproteins, *Tg* triglycerides, *MBP* mean blood pressure.

^a^Two-tailed one-way ANOVA test.

When coronary computed tomography was performed, in 67.5% (52/77) of the patients, calcified atheroma plaques were not found; in 5.2% (4/77) of the patients, a minimal number of calcified plaques was found [0–10 AU]; in 14% (11/77), a low amount [10–100 AU]; in 9.1% (7/77) a moderate amount [100–400 AU]; and in 4% (3/77), a severe amount [> 400 AU]. In 88% of women and in 57% of men, coronary tomography did not show calcified plaques [0–10 AU]. Among all the patients in whom coronary calcium was detected, 91% (20/22) were men (p = 0.004).

The mean risk of suffering a fatal cardiovascular event in the next 10 years calculated using the SCORE system in patients without coronary calcification was 0.52 ± 0.1%; in patients with a coronary artery calcium score of [0–10 AU], it was 1.0 ± 0.7%; in patients with [10–100 AU], it was 1.5 ± 0.45%; in patients with [100 -400 AU], it was 2.0 ± 1.15%; and in those with the highest value [> 400 AU], it was 2.7 ± 0.6% (p < 0.001).

Fifty percent (11/22) of the patients with a coronary calcium score > 10 AU had an IMT > 0.9 mm, and 21% (12/55) of the patients with < 10 UA had an IMT > 0.9 mm (p = 0.015).

Table 2 shows the virological and immunological differences as well as disparities in other inflammatory biomarkers (ultrasensitive PCR and D dimer) between patients with greater and lesser coronary calcification.

The average FMD index of the 77 patients included was 13.02 ± 8.08%. Among the patients with IMT ≥ 0.9, the average FMD was 11.33 ± 6.39%, and among those with IMT < 0.9, the average FMD was 13.24 ± 7.45% (p = 0.26). However, patients who presented a coronary calcium score < 10 AU had an average FMD of 13.53 ± 7.72%, which was significantly higher than those who presented a more severe coronary artery calcium score with a mean FMD of 10.5 ± 5.03% (p = 0.048).

Eighty-six percent (66/77) of the patients included in the study completed the BHI test. Two patients did not complete it due to an inability to complete 20 s of apnoea, and 9 patients did not complete it due to a poor transtemporal ultrasound window.

The average BHI of the population included was 0.89 ± 0.72%. Among the patients with IMT ≥ 0.9, the mean BHI was 0.63 ± 0.39%, and among those with IMT < 0.9, the average BHI was 1.00 ± 0.62% (p = 0.006). Patients who presented a zero or minimal coronary artery calcium score [< 10 AU] presented a BHI of 0.98 ± 0.62%, and those who presented a more severe coronary calcium score had a mean BHI of 0.67 ± 0.44% (p = 0.029). The Pearson correlation between the BHI and the FMD was 0.134 (p = 0.262).

Table 3 shows the relationship between BHI and FMD values and the nadir of CD4 cells, zenith VL, the time since diagnosis and the duration of HAART.

**Table 3:** Clinical and analytical factors associated with endothelial dysfunction measured by BHI and FMD in patients with HIV infection and the relationship with subclinical atherosclerosis.

		Flow Mediated Dilation (n = 77)			Breath Holding Index (n = 66)		
		N	mean ± SD	*P* value^a^	N	mean ± SD	*P* value^a^
**HIV analytical related parameters**							
Nadir CD4 (cells/ μl)	< 200	33	12.21% ± 7.34%		29	0.89% ± 0.57%	
	≥ 200	44	13.63% ± 8.63%	0.44	37	0, 90% ± 0.61%	0.09
Zenith VL (copies/ml)	< 200,000	58	13.17% ± 7.51%		55	0.91% ± 0.58%	
	≥ 200,000	19	11.12% ± 5.91%	0.52	11	0.64% ± 0. 42%	0.06
**HIV clinical related parameters**							
Time of infection (years)	< 20	42	13.63% ± 6.48%		42	0.98% ± 0.66%	
	≥ 20	35	11.16% ± 7.99%	0.16	24	0.75% ± 0.42%	0.09
Time of HAART (months)	< 200	32	13.45% ± 6.42%		28	0.93% ± 0.68%	
	≥ 200	45	11.56% ± 8.06%	0.18	38	0.83% ± 0.43%	0.51
> 1 year from the diagnosis to HAART	Yes	41	11.60% ± 6.76%		34	0.76% ± 0.45%	
	No	36	14.53% ± 8.03%	0.12	32	0.98% ± 0.65%	0.11
**Subclinical atheroesclerosis**							
IMT	< 0,9	54	13.24% ± 7.45%		46	1.00% ± 0.62%	
	≥ 0,9	23	11.33% ± 6.39%	0.26	20	0.63% ± 0.39%	0.006
CACS	< 10 AU	56	13.53% ± 7.72%		44	0.98% ± 0.62%	
	≥ 10AU	21	10.51% ± 5.03%	0.04	22	0.67% ± 0.44%	0.002

*VL* viral load, *HAART* highly active antiretroviral therapy, *IMT* intima media thickness, *CACS* coronary artery calcium score, *SD* standard deviation, *IMT* intima media thickness, *CACs* coronary artery calcium score.

^a^Two-tailed one-way ANOVA test.

The average concentration of CD34 + mononuclear cells was 34.45 ± 65.72 cells/µl, the average concentration of early EPCs was 0.252 ± 0.848 cells/µl, and the concentration of very early EPCs was 0.456 ± 1.15 cells/µl.

Among the patients with a nadir of CD4 T lymphocytes < 350/μl (n = 60) a concentration of CD34 + 309 + 133 + cells of 0.334 ± 0.606 cells/µl was observed, and in patients with a nadir ≥ 350 CD4 (n = 16), the concentration was 0.913 ± 2.21 cells/µl (p = 0.07). The concentration of CD34 + 309 + 133-cells in patients with a CD4 nadir < 350 cells/µl was 0.144 ± 0.218 cells/µl, and among those presenting a CD4 nadir ≥ 350 μl, the concentration was 0.654 ± 1.78 cells/µl (p = 0.03; Table 4).

**Table 4:** Clinical and analytical factors associated with EPCs in patients with HIV infection and the relationship with subclinical atherosclerosis.

		Very Early EPCs			Early EPCs	
		CD34 + 309 + 133 +			CD34 + 309 + 133-	
		n	cells/µl (Mean ± SD)	*P* value^a^	cells/µl (Mean ± SD)	*P* value^a^
**HIV analytical related parameters**						
Nadir CD4 (cells/ μl)	< 350	61	0.335 ± 0.606		0.144 ± 0.218	
	≥ 350	16	0.913 ± 2.214	0.07	0.654 ± 1.786	0.03
Zenith VL (copies/ml)	< 200,000	42	0.554 ± 1.30		0.313 ± 0.963	
	≥ 200,000	35	0.123 ± 0.17	0.20	0.059 ± 0.076	0.31
**HIV clinical related parameters**						
Time of infection (years)	< 20	32	0.318 ± 0.578		0.156 ± 0.245	
	≥ 20	45	0.666 ± 0.245	0.28	0.397 ± 0.284	0.33
Time of HAART (months)	< 200	41	0.291 ± 0.396		0.152 ± 0.220	
	≥ 200	36	0.682 ± 1.711	0.21	0.381 ± 1.281	0.34
> 1 year from diagnosis to HAART	Yes	41	0.308 ± 0.598		0.373 ± 1.239	
	No	36	0.639 ± 1.583	0.254	0.153 ± 0.237	0.31
**Subclinical atheroesclerosis**						
IMT	< 0,9	54	0.460 ± 1.285		0.298 ± 1.000	
	≥ 0,9	23	0.445 ± 0.787	0.95	0.143 ± 1.286	0.30
CACS	< 10 AU	56	0.388 ± 0.640		0.176 ± 0.241	
	≥ 10AU	21	0.622 ± 1.913	0.58	0.437 ± 1.540	0.44

*EPCs* endothelial progenitor cells, *VL* viral load dl, *HAART* highly active antiretroviral therapy, *SD* standard deviation, *IMT* intima media thickness, *CACs* coronary artery calcium score.

^a^Two-tailed one-way ANOVA test.

The relationship between the concentration of EPCs and the chronology of the infection is shown in Table 4.

**Figure 2:**
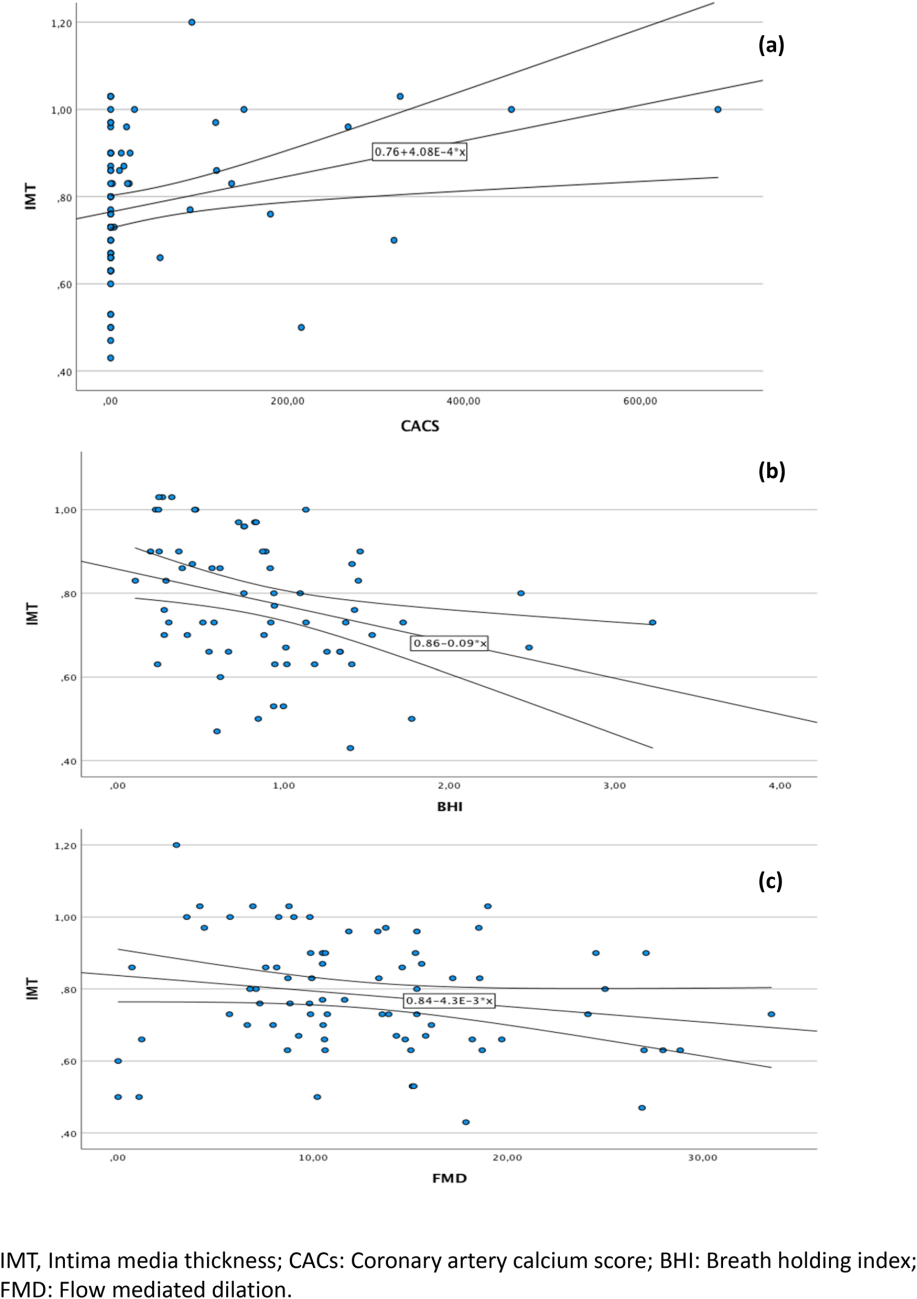
Correlation between IMT and other subclinical atherosclerosis and endothelial dysfunction tests. (**a**) IMT versus CAC scatter plots with 95% CI linear fit. (**b**) IMT versus BHI scatter plots with 95% CI linear adjustment. (**c**) IMT versus FMD scatter plots with linear 95% CI adjustment.

**Figure 3:**
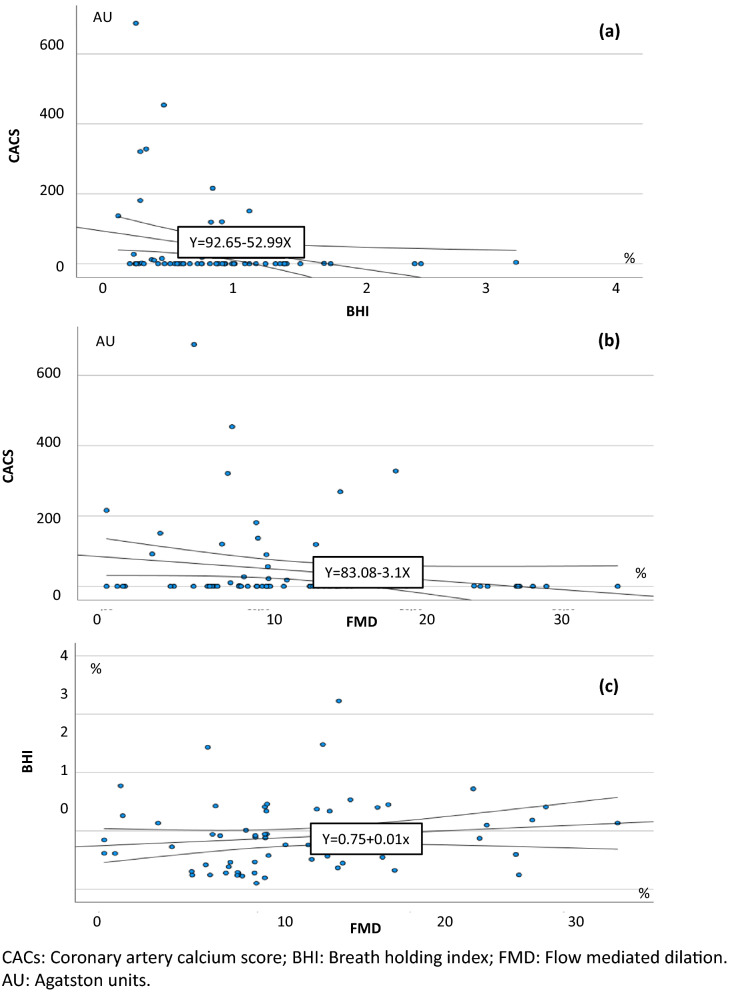
Correlation between CACs and the endothelial dysfunction test. (**a**) CACs versus IHC scatter plots with linear 95% CI adjustment. (**b**) CACs versus FMD scatter plots with linear 95% CI adjustment. (**c**) BHI versus FMD scatter plots with linear 95% CI adjustment.

Age and sex were used as covariates in the multivariate statistical analysis whenever significant associations were found between the variable under study and age or sex in the univariate analysis. No significant associations were found (Tables 5 and 6). The correlations between subclinical atherosclerosis and evidence of endothelial dysfunction are shown in Figs. 2 and 3.

**Table 5:** Correlation between HIV-related analytical parameters and subclinical atherosclerosis and endothelial dysfunction. Unadjusted model and sex- and age-adjusted model.

	CD4		Nadir CD4		Zenith VL	
	r	*P* value	r	*P* value	r	*P* value
**Unajusted model**^**a**^						
Subclinical atheroesclerosis						
IMT	0.11	0.33	0.01	0.94	0.19	0.11
CACs	0.06	0.59	0.14	0.24	0.01	0.91
Endothelial dysfunction						
BHI	0.20	0.12	− 0.07	0.59	− 0.04	0.76
FMD	0.04	0.76	0.03	0.78	− 0.21	0.06
Very Early EPCs	− 0.04	0.71	0.07	0.56	− 0.07	0.54
EPCs	0.02	0.85	0.15	0.32	− 0.05	0.65
**Sex and age ajusted model**^**a**^						
Subclinical atheroesclerosis						
IMT	0.06	0.54	0.29	0.77	0.13	0.21
CACs	0.02	0.86	0.10	0.38	0.08	0.48
Endothelial dysfunction						
BHI	0.11	0.36	− 0.19	0.10	− 0.04	0.73
FMD	− 0.05	0.65	0.02	0.19	− 0.21	0.09
Very Early EPCs	− 0.05	0.67	0.07	0.62	− 0.09	0.41
EPCs	0.02	0.85	0.12	0.27	− 0.08	0.48

*VL* viral load, *IMT* intima media thickness, *CACs* coronary artery calcium score, *BHI* breath holding index, *FMD* flow mediated dilation, *EPC* endothelial progenitor cells.

^a^Multiple linear regression models.

**Table 6:** Correlation between HIV-related analytical parameters and subclinical atherosclerosis and endothelial dysfunction. Unadjusted model and sex- and age-adjusted model.

	Time of infection		Time of HAART	
	r	*P* value	r	*P* value
**Unajusted model**^**a**^				
Subclinical atheroesclerosis				
IMT	0.02	0.89	0.076	0.51
CACs	0.02	0.87	0.03	0.79
Endothelial dysfunction				
BHI	− 0.13	0.29	− 0.04	0.71
FMD	− 0.14	0.12	− 0.15	0.18
Very Early EPCs	0.18	0.12	0.14	0.23
EPCs	0.14	0.21	0.08	0.46
**Sex and Age ajusted model**^**a**^				
Subclinical atheroesclerosis				
IMT	0.12	0.27	0.05	0.63
CACs	0.01	0.97	0.04	0.97
Endothelial dysfunction				
BHI	− 0.02	0.84	0.04	0.70
FMD	− 0.17	0.15	− 0.15	0.19
Very Early EPCs	0.16	0.17	0.14	0.23
EPCs	0.11	0.34	0.08	0.48

*HAART* high active antiretroviral activity, *IMT* intima media thickness, *CACs* coronary artery calcium score, *BHI* breath holding index, *FMD* flow mediated dilation, *EPC* endothelial progenitor cells.

^a^Multiple linear regression models.

## Discussion

It has been shown that HIV infection is associated with an increased cardiovascular risk^1,2,4^. Therefore, new tests are needed to allow an early diagnosis. To our knowledge, our study is the first to evaluate markers of endothelial dysfunction, such as EPCs, BHI and FMD, and markers of subclinical atherosclerosis, such as IMT and the coronary calcium score, in the same patients with HIV infection and to study their relationship with HIV-related parameters, such as VL and CD4 cells. Each of the diagnostic tests is discussed below.

In this study, as in other publications, the IMT patients with HIV infection is associated with classic cardiovascular factors such as age and sex, as in the general population^7–10^. Regarding the relationship of IMT and immune status, we found a trend towards a lower CD4/CD8 ratio among patients with IMT ≥ 0.9 mm, although the difference did not reach statistical significance. Similar findings have been published previously^8^. Furthermore, we observed that patients with pathological IMT had a lower value of CD4 cells at the time of study inclusion. Previous studies have observed that patients with a CD4 cell value < 200 have a higher IMT and that the progression over time of IMT is faster than in those with normal immunological status^9,26^.

Regarding the coronary calcium score, we found that patients whose calcium score was > 100 AU not only had a lower CD4 cell value at the time of study inclusion and a lower CD4 cell nadir, also had a significantly higher zenith VL.

The findings on the association between coronary calcium quantification and HIV-related parameters is controversial. Chow et al.^11^, in a study with methodologic similar to ours, did not observe an association between lymphocyte subpopulations and VL with coronary calcium score in patients with HIV infection. This fact is probably related to a greater prevalence of classical vascular risk factors in the included population, since the patients were older and a large percentage of them were smokers (63%)^11^. However, in other studies with a lower percentage of smokers, whose population was more similar to ours, the progression over time of coronary calcium score was associated with the VL of the patients and with the number of CD4 cells^12,13^.

We did not find a significant association between FMD and the different parameters related to HIV infection, although there was a tendency towards a greater FMD among patients with a worse immunological and virological status and a longer infection time. The findings on this association are also controversial. While a study, similar to this one, found no differences in FMD in the different subgroups of patients with HIV infection^14^, other work has found an association between FMD and HIV-related parameters, and the FMD values lower than in our study^27^. This may be related to the fact that the patients with HIV infection included in that study did not receive HAART and that we do not know if the methodology for measuring FMD was the same as in our study. Another study compared FMD in HIV patients receiving HAART and naive patients. In the naive subgroup, the vascular reactivity was greater than in the group of patients receiving treatment, to the trend observed in our study^15^. These findings may be related to poorer lipidic control and a higher percentage of smokers among patients already receiving HAART.

HIV infection has been associated with lower cerebral reactivity without a relationship between BHI and CD4 cells^16^ and with a trend towards higher cerebral vasoreactivity for each additional year of viral suppression^17^. These data are also in accordance with our findings, as patients with a high VL had a worse BHI. In fact, in our study, patients who presented a higher zenith VL (≥ 200,000 copies/ml) had a lower cerebral vascular reactivity measured by BHI than those with zenith CV < 200,000 copies/ml, which was just over the limits of statistical significance. We did not find an association between BHI and CD4 cell values, and those patients with a longer time since HIV diagnosis and those who did not receive antiretroviral treatment for long periods of time also had a worse BHI. Patients with a pathological IMT and a calcium score > 100 UA had a lower BHI. Therefore, those patients who achieved higher viral loads and who remained off HAART for longer since HIV diagnosis may have worse endothelial dysfunction as measured by BHI.

When EPCs were determined, patients with a lower CD4 cell nadir and with a higher zenith VL had a lower blood concentration of early and very early EPCs, although no statistically significant differences were reached. Patients with more time on HAART, more time of infection and more time without treatment had a minor concentration of EPCs. There are few studies that have determined EPCs in patients with HIV infection. In Seang et al.’s study^19^, EPCs were measured in 57 HIV-positive men, and the concentration was lower than in our study, with more patients with no cells detected. This fact may be related to a higher prevalence or to classic cardiovascular risk factors in their patients, who were older and in whom dyslipidaemia and diabetes were more frequent. No association was found between EPCs and HIV-related parameters.

Papassavas et al.^20^ measured the same type of EPCs as us, and a direct association between early EPCs and CD4 cells was observed. However, in the rest of the studies, this relationship was not found with VL^23,24^. In all the studies that have measured EPCs in patients with HIV infection except in that of Costinuk et al.^21^, patients who smoked were included, and it is difficult to draw conclusions if we take into account that nicotine alters the proliferation of EPCs. The lack of a consensus definition in other articles on EPCs^22–24^ and the heterogeneity of classic cardiovascular risk factors in their populations make it difficult to draw conclusions.

Our study has several limitations. First, the small sample size decreased our power to detect relationships between diagnostic tests and HIV-related parameters. However, the careful selection of patients without cardiovascular risk factors and non-smoking patients made it possible to eliminate confounding factors and to relate VL and CD4 cells with some of the selected tests.

The lack of a control group of patients without HIV infection could be considered a limitation. However, since the higher incidence of atherosclerosis among HIV-infected patients has been demonstrated in previous studies^3–6^, and our aim was to study which clinical and analytical parameters related to HIV infection were associated with subclinical atherosclerosis and endothelial dysfunction, we compared the clinical (time on treatment, time of infection) and analytical parameters (CD4 cells and CD4 nadir, zenith VL) of HIV patients with alterations in the diagnostic tests for atherosclerosis with those without such alterations, considering this latter group as a control group.

## Conclusions

In HIV-positive patients, low CD4 cell levels and high VL are associated with an increased risk of subclinical atherosclerosis despite having a low SCORE index. We did not find a significant association between endothelial function and parameters related to HIV infection, such as CD4 cells or VL. HIV patients with lower CD4 cells may have fewer early EPCs. In HIV-infected patients, despite a low SCORE index, if they had a high zenith VL and a low current nadir or CD4 cell concentration, diagnostic tests (IMT or coronary artery calcium score) could be indicated for the diagnosis of subclinical atherosclerosis, which would allow primary prevention. Further studies are needed to introduce new techniques for the diagnosis of subclinical cardiovascular disease and endothelial dysfunction into clinical practice, but the patients who benefit most from them are likely those with the worst virological and immunological history.

## Patients and methods

A prospective cohort study was carried out at the Marques de Valdecilla University Hospital from June 1, 2015, to May 31, 2018, which included patients with HIV infection with more than 5 years of HAART and low cardiovascular risk according to the SCORE index and without previous cardiovascular events. The following exclusion criteria were considered: patients who were not virally suppressed (> 20 copies/ml), smokers or former smokers for the 15 years prior to inclusion, patients who had less than 5 years of HAART and patients suffering from systemic inflammatory diseases (connective tissue diseases, vasculitis, inflammatory bowel diseases or others other than the study groups). Arterial hypertension or dyslipidaemia were not considered exclusion criteria when there was no damage to the target organ.

Screening was performed on 1332 patients. Fifty-one men and 26 women met the inclusion and exclusion criteria and agreed to enter the study. All patients included were followed up until September 2020. During the follow-up, 1 patient suffered an acute myocardial infarction, and another suffered a stroke. Two patients died from a neoformative process. No patient died from a cardiovascular event.

Data were collected by a direct interview with the patient and a review of their medical records. The following HIV-related parameters were registered in a database designed specifically for this purpose: CD4 cell concentration, nadir CD4 cells and zenith VL. Additionally, D dimer, erythrocyte sedimentation rate, ultrasensitive C-reactive protein, lipid profile (total cholesterol, cholesterol bound to high-density lipoproteins (HDLc), cholesterol bound to low-density lipoproteins (LDLc), triglycerides and complete blood count) were recorded. All studies were performed in subjects after an overnight fast (> 8 h), without exposure to vasoactive medications and caffeine intake for at least the previous 24 h and were performed in a temperature-controlled room (22 °C). Measurement of blood pressure and blood draws were performed at the time of ultrasonographic study.

### Definitions and methodology of diagnostic tests

For the measurement of IMT, the carotid territory was explored in a standardized way. The patient was placed in a supine position and using high-resolution B-mode ultrasound with a 10 MHz linear transducer, the IMT was measured at 1 cm below the carotid bulb in the far wall of the vessel. In addition, the presence or absence of atheroma plaques was collected^7^.

All patients underwent computed tomography imaging of coronary arteries using a 32-slice multidetector scanner to determine the coronary calcium score. This is the sum of the calcium scores (measured as Agatston scores of all calcifications) in the left main coronary artery, left anterior descending artery, left circumflex coronary artery, right coronary artery, and posterior descending artery. Patients were stratified into 4 groups: 0 (normal), 1–100 (low to moderate cardiovascular risk), 101–400 (moderate to high cardiovascular risk), and > 400 (high cardiovascular risk)^28^.

The recommendations of the International Brachial Artery Reactivity Task Force^29^ were followed to calculate FMD. The image of the brachial artery was obtained above the antecubital fossa in the longitudinal plane. To create a flow stimulus, a blood pressure cuff was inflated on the proximal forearm, approximately 50 mmHg above the systolic blood pressure of the patient for 5 min. Approximately 60 s after withdrawal of the cuff, measurements of the artery diameter were made again.

$${\text{FMD}} = [({\text{post - occlusiondiameter }} - {\text{basal diameter}})/{\text{basal diameter}}] \, \times \, 100.$$

For the calculation of the BHI, it is necessary to locate the middle cerebral artery, which is approximately 50 mm from the surface through the transtemporal window. Once the basal average velocity was obtained, the patients were asked to hold their breath for 30 s to obtain a new velocity record (Vbh). With the data obtained, the BHI was calculated with the following formula^30^:

$$BHI \, = \, \left[ {\left( {Vbh \, - \, Vb} \right)/Vb} \right) \, \times \, Duration \, of \, apnoea \, \left( s \right)] \, \times 100.$$

The number of EPCs was measured using flow cytometry on whole blood samples. Blood was collected in two sodium heparin tubes in the fasting state. Peripheral blood EPCs were quantified using a previously described protocol^18^. Antibodies against CD-34 PE, KDR-APC, CD62E-FITC, CD34-FITC, KDR-APC, and CD 133-PE were added to two different tubes. The populations under study were separated in a BD FACS Aria 1 model flow cytometer, and the results obtained were analysed with FACSDiva Software V5.03 ModFit V3.0. The populations identified for each of the samples were the following:

$${\text{stem haematopoietic cells }}{-}{\text{ CD34}} + ;{\text{ very early EPCs }}{-}{\text{ CD34}} + {\text{ KDR}} + {\text{ CD133}} + ;{\text{ and early EPCs }}{-}{\text{ CD34}} + {\text{ KDR}} + {\text{ CD 133}} - .$$

### Statistical analysis

Quantitative variables are expressed as the mean and standard deviation (SD); qualitative variables are expressed as frequency and percentage. Statistical analysis was performed using a two-tailed χ2 test and Fisher’s exact test or an analysis of variance test (ANOVA) (one way and two-way ANOVA), as appropriate in each case. The association between continuous variables was assessed using the Pearson correlation coefficient (r) and a multiple linear regression analysis. A two-tailed p < 0.05 was considered statistically significant. Data were analysed using SPSS package v19.0 (SPSS Inc., Chicago, IL) and Stata statistical software (Release 11.0, Stata Corporation, College Station, TX).

### Ethical approval and informed consent

The study was performed in accordance with the Declaration of Helsinki. The protocol was reviewed and approved by the Clinical Research Ethics Committee of Cantabria (Ref: 2015.09) according to local standards. Informed consent was obtained from each patient.
